# The Systematic Treatment of Nerve Prostration and Hysteria

**Published:** 1883-04

**Authors:** 


**The Systematic Treatment of Nerve Prostration and Hysteria.** By W. S. PLAYFAIR, M. D., F. R. C. P., Professor of Obstetric Medicine in King's College, etc. Philadelphia: Henry C. Lea's Son & Co. 1883.

Dr. Playfair aims, in this little work, to illustrate the system introduced to the notice of the profession by Dr. Weir Mitchell in the treatment of nerve-tire connected with uterine disease. The influence of rest and seclusion, massage, electricity, diet and regimen are very clearly illustrated in the report of cases of this type of disease. The book is exceedingly readable, and is deserving of the attention of the profession.

